# ShinyGPAS: interactive genomic prediction accuracy simulator based on deterministic formulas

**DOI:** 10.1186/s12711-017-0368-4

**Published:** 2017-12-20

**Authors:** Gota Morota

**Affiliations:** Department of Animal Science, University of Nebraska-Lincoln, PO Box 830908, Lincoln, NE 68583-0908 USA

## Abstract

**Background:**

Deterministic formulas for the accuracy of genomic predictions highlight the relationships among prediction accuracy and potential factors influencing prediction accuracy prior to performing computationally intensive cross-validation. Visualizing such deterministic formulas in an interactive manner may lead to a better understanding of how genetic factors control prediction accuracy.

**Results:**

The software to simulate deterministic formulas for genomic prediction accuracy was implemented in R and encapsulated as a web-based Shiny application. Shiny genomic prediction accuracy simulator (ShinyGPAS) simulates various deterministic formulas and delivers dynamic scatter plots of prediction accuracy versus genetic factors impacting prediction accuracy, while requiring only mouse navigation in a web browser. ShinyGPAS is available at: https://chikudaisei.shinyapps.io/shinygpas/.

**Conclusion:**

ShinyGPAS is a shiny-based interactive genomic prediction accuracy simulator using deterministic formulas. It can be used for interactively exploring potential factors that influence prediction accuracy in genome-enabled prediction, simulating achievable prediction accuracy prior to genotyping individuals, or supporting in-class teaching. ShinyGPAS is open source software and it is hosted online as a freely available web-based resource with an intuitive graphical user interface.

## Background

Prediction of breeding values from high-dimensional single nucleotide polymorphisms is a primary interest in quantitative genetics [1–3]. This is particularly true for the application of genomic selection in animal and plant breeding programs, where genetic improvement of agricultural species relies on the performance of a model to predict unknown breeding values, also known as prediction accuracy. Here prediction accuracy is defined as the correlation between true and predicted genomic values. A deterministic formula such as the one proposed by Daetwyler et al. [4] highlights the relationship between prediction accuracy and potential factors that influence prediction accuracy. In general, deterministic formulas compute the expected predictive correlation (or squared prediction accuracy $R^2$) on the basis of a number of factors that are potentially useful to assess prediction accuracy before performing computationally demanding cross-validation (CV). It also allows us to decide the optimal design for reference populations (e.g., reference population size) to achieve a desired level of accuracy in selection candidates. Not only theoretical derivations of deterministic formulas but also their applications are active research areas. For instance, Brard and Ricard [5] recently performed comparison and meta-analysis of deterministic formulas. Erbe et al. [6] inferred parameters that influence prediction accuracy in deterministic formulas via maximum likelihood. Collectively, these studies have shed new light on alternative aspects of factors influencing predictive performance that may not be obvious from empirical genome-enabled prediction analysis based on CV.

In particular, visualizing such deterministic formulas may lead to a better understanding of how genetic factors control prediction accuracy. Typically, visualization involves generating a static two-dimensional graph, where the y-axis is the genomic prediction accuracy and the x-axis is one of the factors influencing prediction accuracy, while keeping the other factors constant. Given that this type of static graph is a snapshot of a complex dynamic system, if users want to change parameters, they need to re-type and re-execute the code. To provide an overview of the whole landscape of genomic prediction simulation, we need an efficient visualization tool that is capable of generating interactive as well as dynamic graphs. The objective of this article is to describe a Shiny-based web application called Shiny genomic prediction accuracy simulator (ShinyGPAS), which produces interactive graphs and offers an intuitive graphical user interface (GUI) for simulating genomic prediction accuracy based on deterministic formulas.

## Software description

### Overview of software architecture

**Fig. 1:**
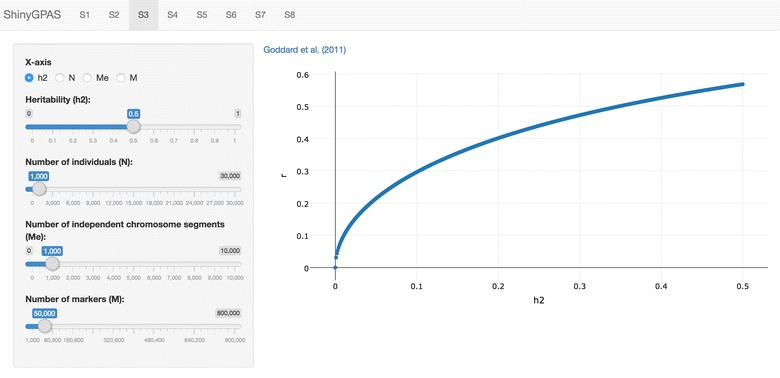
Each deterministic formula is implemented in a tab on the top. The y-axis is the prediction accuracy and the x-axis is one of the parameters. Parameters such as heritability, the number of individuals, the number of independent chromosome segments, and the number of markers can be set by the user

ShinyGPAS is implemented entirely in R, which is an open source programming language and environment for performing statistical computing and data visualization [7]. The GUI is provided by the shiny R package [8], a web application framework for R. ShinyGPAS is a Shiny application that leverages R and the shiny package to construct an intuitive framework for deterministic formulas using dynamic interaction and visualization. The ShinyGPAS user interface is shown in Fig. 1. Although ShinyGPAS is R-based software, it does not require users to be familiar with the programming language or download the software on a local computer. The underlying R code is encapsulated by Shiny and offered as cohesive web-based software to be usable solely by mouse navigation in a web browser. This increases accessibility to the software, especially for users with less R programming experience. ShinyGPAS is deployed through the cloud-based shinyapps.io platform for hosting Shiny web applications (https://www.shinyapps.io/).

### Deterministic formulas

ShinyGPAS currently delivers eight simulators based on deterministic formulas described in (a) Daetwyler et al. [4, 9], (b) Goddard [10], (c) Goddard et al. [11], (d) Rabier et al. [12], (e) Rabier et al. [12], (f) de los Campos et al. [13], (g) Karaman et al. [14] and (h) Wientjes et al. [15]. The first seven formulas predict accuracy within populations whereas the last one is designed for multipopulation scenarios, including multi-environment and multitrait. Deterministic formulas are functions derived from the combinations of the number of individuals in a reference set, the number of independent chromosome segments underlying the trait, the effective population size, the number of markers, the proportion of genetic variance explained by the molecular markers, and heritability. Shiny-based interactive application offers the implementation of dynamic deterministic formulas, allowing to evaluate the simultaneous impact of all the parameters described above on the prediction accuracy. A user can click a link located within each deterministic formula simulator to access original journal articles. Below are deterministic formulas currently implemented in ShinyGPAS.

- Daetwyler et al. [4, 9] developed the first deterministic formula that computes the prediction accuracy of additive genomic values. The formula was derived by treating genetic markers as fixed with the following assumptions: (a) independence of quantitative trait loci (QTL), (b) regression of phenotypes on genotype one locus at a time with $\sigma ^2_{\epsilon } = 1$ and $\sigma ^2_g + \sigma ^2_{\epsilon } = 1$ ($\sigma ^2_{g}$ and $\sigma ^2_{\epsilon }$ are the genetic and residual variances, respectively), (c) identical accuracy of QTL effect size estimates across QTL allele frequencies and (d) perfect linkage disequilibrium (LD) between marker and QTL pairs.
  $$\begin{aligned} r = \sqrt{\frac{N h^2}{N h^2 + M_e} }, \end{aligned}$$
  where *N* is the number of individuals in the reference population, $h^2$ is the heritability, and $M_e$ is the number of independent chromosome segments.
- Goddard [10] developed an alternative formula by treating markers as random and assuming complete LD between marker and QTL pairs. The QTL effects were assumed to be sampled from a normal distribution. In addition, the equation assumes that QTL with high minor allele frequencies have more accurate effect size than QTL with low minor allele frequencies.
  $$\begin{aligned} r = \sqrt{1 - \frac{\lambda }{2N\sqrt{\alpha }} \ln \left( \frac{1 + \alpha + 2\sqrt{\alpha }}{1 + \alpha - 2\sqrt{\alpha }}\right) }, \end{aligned}$$
  where $\lambda$ is $M_e/(h^2\ln (2N_e))$, $\alpha$ is $1 + 2(M_e/Nh^2\ln (2N_e))$, and $N_e$ is the effective population size. The definition of $\lambda$ was adopted from Hayes et al. [16, 17]. Note that here $M_e$ and $N_e$ are related because $M_e$ can be expressed as a function of $N_e$ [10].
- Goddard et al. [11] extended the equations in [4, 9, 10] so that the deterministic formula accounts for incomplete LD between markers and QTL. This equation also accounts for the fact that the number of markers is finite.
  $$\begin{aligned} r = \sqrt{b \frac{Nbh^2/M_e}{1 + Nbh^2/M_e}}, \end{aligned}$$
  where $b = M/(M + M_e)$ is the proportion of genetic variance explained by the markers and *M* is the number of markers. Note that when *b* is equal to 1, this deterministic formula is identical to that of Daetwyler et al. [4, 9].
- Rabier et al. [12] developed a deterministic formula by relaxing the assumption of $\sigma ^2_{\epsilon } = 1$ and $\sigma ^2_g + \sigma ^2_{\epsilon } = 1$ in Daetwyler et al. [4]. This formula can be applied with any value of $\sigma ^2_g$ and $\sigma ^2_{\epsilon }$.
  $$\begin{aligned} r = \sqrt{\frac{h^2/(1-h^2)}{M_e/N + h^2/(1-h^2)}}. \end{aligned}$$
  Moreover, under the ridge regression best linear unbiased prediction framework, $M_e/N$ is equal to $\mathbb{E}(||\mathbf{x}'_{n_{\text{TRN} + 1}} \mathbf{X}'\mathbf{V}^{-1} ||^2)$, where $\mathbf{x}'_{n_{\text{TRN} + 1}}$ is the vector of markers belonging to the testing set individual, $\mathbf{X}$ is the training set allele content matrix, $\mathbf{V} = \mathbf{XX}' + \lambda \mathbf{I}$, $\lambda$ is the regularization parameter, and $||.||^2$ is the squared norm. Therefore, an alternative form of prediction accuracy when fitting the all markers simultaneously in a high-dimensional setting [18] is obtained by replacing $M_e$ with $N \cdot \mathbb{E}(||\mathbf{x}'_{n_{\text{TRN} + 1}} \mathbf{X}'\mathbf{V}^{-1} ||^2)$ [12].
  $$\begin{aligned} r = \sqrt{\frac{h^2/\left( 1-h^2\right) }{\mathbb{E}\left( ||\mathbf{x}'_{n_{\text{TRN} + 1}} \mathbf{X}'\mathbf{V}^{-1} ||^2\right) + h^2/\left( 1-h^2\right) }}. \end{aligned}$$
  Note that if we can assume individuals in training and testing sets were sampled from the same population, $\hat{\mathbb{E}}(||\mathbf{x}'_{n_{\text{TRN} + 1}} \mathbf{X}'\mathbf{V}^{-1} ||^2) \le 1$ then, $N * \hat{\mathbb{E}}(||\mathbf{x}'_{n_{\text{TRN} + 1}} \mathbf{X}'\mathbf{V}^{-1} ||^2)$ is bounded by *N*.
- de los Campos et al. [13] developed an equation that yields a theoretical upper limit for the achievable accuracy. This formula was motivated by the assumption that the patterns of allele sharing between markers and causal loci are different. Under the genomic best linear unbiased prediction framework
  $$\begin{aligned} r = \sqrt{ \left[ 1 - (1 - b)^2\right] h^2 }, \end{aligned}$$
  where *b* is the average regression coefficient of the marker-based genomic relationships on genomic relationships at QTL. This deterministic formula does not rely on $M_e$, which is difficult to infer from data.
- Karaman et al. [14] expressed the deterministic equations of Daetwyler et al. [4, 9] and Goddard et al. [11] in terms of the correlation between phenotypes and estimated breeding values. Note that this equation and also that of de los Campos et al. [13] can be viewed as the measure of prediction accuracy.
  $$\begin{aligned} r = \sqrt{ h^2_M \left[ \frac{N h^2_M}{N h^2_M + M_e} \right] }, \end{aligned}$$
  where $h^2_M$ is the genomic heritability, which is the proportion of phenotypic variance that is explained by regression on markers.
- Wientjes et al. [15] developed the deterministic formula that combines two populations A and B to predict prediction accuracy in population C. This can be used for multipopulation genomic prediction scenarios.
  $$\begin{aligned} r&= \sqrt{ \begin{bmatrix} b_{AC} r_{G_{AC}} \sqrt{\frac{h^2_A}{M_{e_{A,C}}} }&b_{BC} r_{G_{BC}} \sqrt{\frac{h^2_B}{M_{e_{B,C}}}} \end{bmatrix}} \\&\quad \times \sqrt{\begin{bmatrix} \frac{h^2_A}{M_{e_{A,C}}} + \frac{1}{N_A}&r_{G_{AB}} \sqrt{\frac{h^2_A h^2_B}{M_{e_{A,C}} M_{e_{B,C}} } } \\ r_{G_{AB}} \sqrt{\frac{h^2_A h^2_B}{M_{e_{A,C}} M_{e_{B,C}} } }&\frac{h^2_B}{M_{e_{B,C}}} + \frac{1}{N_B} \end{bmatrix}^{-1}} \\&\quad \times \sqrt{\begin{bmatrix} b_{AC} r_{G_{AC}} \sqrt{\frac{h^2_A}{M_{e_{A,C}}}} \\ b_{BC} r_{G_{BC}} \sqrt{\frac{h^2_B}{M_{e_{B,C}}}} \end{bmatrix}}, \end{aligned}$$
  where $b_{AC}$is the square root of the proportion of the genetic variance in predicted population C explained by the markers in training population A, $r_{G_{AC}}$ is the genetic correlation between populations A and C, $h^2_A$ is heritability in population A, $b_{BC}$ is the square root of the proportion of the genetic variance in predicted population C explained by the markers in training population B, $r_{G_{BC}}$ is the genetic correlation between populations B and C, $h^2_B$ is heritability in population B, $N_A$ is the number of individuals in population A, $N_B$ is the number of individuals in population B, $r_{G_{AB}}$ is the genetic correlation between populations A and B, and $M_{e_{A,C}}$ and $M_{e_{B,C}}$ are the effective numbers of chromosome segments shared between populations A and C, and B and C, respectively. Note that the *b* values of Goddard et al. [11] are the squares of the *b* values in the above equation.

Prediction of genomic values is a challenging task and there is no universally best deterministic formula that accounts for all potential factors. Therefore, we will continue adding newly developed deterministic formula to ShinyGPAS.

### Program input

A typical workflow starts from selecting one of the tab panels on the top (Fig. 1) and then moving to a preferred deterministic formula simulator. Each deterministic formula captures a different aspect of the genotype–phenotype map in the context of genomic prediction accuracy. Thus, navigate through interactively visualized deterministic formulas may highlight the common patterns as well as differences among them. A suite of available parameters such as $h^2$, $h^2_M$ *N*, $M_e$, $N_e$, *M*, and *b* are located in the sidebar panel. Shiny slider provides possible input values that can be chosen from pre-defined ranges. Users can pick a preferred value by a simple mouse navigation. A radio button located on the top offers possible options for factors that influence prediction accuracy to be used to determine the x-axis. The Shiny reactive expressions are used in ShinyGPAS to efficiently cache results and ease computational burden to ensure high speed of processing during an interactive session.

### Program output

Rendering interactive graphs from deterministic formulas are achieved by the plotly R package [19]. The main engine plotly.js, which is built on top of JavaScript and the visualization library D3.js, was used to create a scatter plot. The y-axis is pre-fixed with prediction accuracy (*r*). Users can choose the x-axis from one of the parameters such as $h^2$, $h^2_M$, *N*, $M_e$, $N_e$, *M*, or *b*. A scatter plot is dynamically updated when users vary slider input values of factors that influence prediction accuracy. The plotly.js generates a scatter plot with a toolbar coupled with useful zooming in and zooming out capabilities. Also, hovering the mouse pointer over a specific point of plot shows the exact values of x and y axes. A multipopulation genomic prediction simulation is enabled by the plotly 3D scatter plot functionality, where x and y axes take parameters from two training populations and the z-axis shows prediction accuracy. Rotating the 3D scatter plot is possible around all x, y and z axes to inspect prediction accuracy from different surfaces. In addition, the toolbar provides features such as a download button, box select, lasso select, autoscale, reset, and toggle spike lines for interactivity. ShinyGPAS is available at: https://chikudaisei.shinyapps.io/shinygpas/.

## Conclusions

A Shiny application has great potential to deliver interactive data analysis and visualization in a web browser. Yet there is limited application of this type of tool in breeding and quantitative genetics. The Shiny framework allows users to convert deterministic formulas of genomic prediction accuracy into interactive graphics in an engaging and straightforward manner. ShinyGPAS can be used for interactive exploration of potential factors that influence prediction accuracy in genome-enabled prediction, simulation of achievable prediction accuracy prior to genotyping individuals, or supporting in-class teaching. The ShinyGPAS source code has been made publicly available on GitHub: https://github.com/morota/ShinyGPAS.
